# Discovery of *Fissidens pokhrensis* (Fissidentaceae) in China and an updated key to Chinese species with semilimbate leaves and papillose or mammillose laminal cells

**DOI:** 10.3897/phytokeys.276.187004

**Published:** 2026-06-09

**Authors:** Zi-Qi Liu, An-Ran Ke, Boon-Chuan Ho, Shun-Li Wang, Yu-Mei Wei, Wen Ye

**Affiliations:** 1 School of Life Sciences, Xiamen University, Xiamen 361102, China Guangxi Institute of Botany, Guangxi Zhuang Autonomous Region and Chinese Academy of Sciences Guilin China; 2 Singapore Botanic Gardens, National Parks Board, 1 Cluny Road, Singapore 259569, Singapore School of Life Sciences, Xiamen University Xiamen China; 3 Department of Biological Sciences, National University of Singapore, 14 Science Drive 4, Singapore 117543, Singapore Department of Biological Sciences, National University of Singapore Singapore Singapore; 4 School of Life Science, Guizhou Normal University, Guiyang 550025, China School of Life Science, Guizhou Normal University Guiyang China; 5 Guangxi Key Laboratory of Plant Conservation and Restoration Ecology in Karst Terrain, Guangxi Institute of Botany, Guangxi Zhuang Autonomous Region and Chinese Academy of Sciences, Guilin 541006, China Singapore Botanic Gardens, National Parks Board Singapore Singapore

**Keywords:** Biodiversity hotspots, bryophyte, epiphyte, Gaoligong Mountains, subg. *Polypodiopsis* sect. *Antennidens*

## Abstract

*Fissidens* is one of the most diverse genera of mosses in China. Most members of this genus are terrestrial, with a few preferring tree trunks as substrates. *Fissidens pokhrensis* is newly discovered in China, occurring at the base of a tree in Yunnan Province. Morphological descriptions and photographs of this species are provided. In addition, an updated identification key is presented for Chinese species of *Fissidens* characterized by semilimbate leaves and papillose or mammillose laminal cells, including *F. pokhrensis*.

## Introduction

*Fissidens* Hedw. (Hedwig 1801), the sole genus of the moss family Fissidentaceae, comprises an estimated 450–500 species worldwide (Pursell 1982, 2007). Members of this family are distinguished by their uniquely structured leaves, each consisting of paired vaginant laminae, an apical lamina, and a dorsal lamina, arranged distichously in a single plane to form dorsiventrally flattened, feather-like shoots.

In recent years, molecular phylogenetic studies have continuously reshaped the infrageneric classification of the genus *Fissidens*. Suzuki et al. (2018) established the first comprehensive infrageneric classification based on the chloroplast *rbcL* and *rps4* genes to recognize three subgenera: subg. *Pachyfissidens*, subg. *Neoamblyothallia* (sections *Neoamblyothallia* and *Crispidium*), and subg. *Fissidens* (sections *Fissidens*, *Polypodiopsis*, *Aloma*, *Areofissidens*, and *Semilimbidium*). Subsequently, Budke et al. (2023) expanded this genomic scope by employing target-capture sequencing of 400 loci, providing high-resolution phylogenomic trees to test existing systems. Their results supported the monophyly of subgenera *Aloma* and *Octodiceras* (sensu Pursell and Bruggeman-Nannenga 2004), as well as subg. *Pachyfissidens*, subg. *Neoamblyothallia* sect. *Neoamblyothallia*, and subg. *Fissidens* sect. *Areofissidens* (sensu Suzuki et al. 2018). However, all other subgenera and sections defined by these two systems were found to be non-monophyletic. These findings underscore the evolutionary lability of key morphological traits, suggesting that broader taxon sampling and more extensive molecular data are required to further refine and improve the infrageneric classification system of the genus.

Located in western Yunnan, China, the Gaoligong Mountains extend approximately 600 km along the western margin of the Hengduan Mountains (Zhou et al. 2023). Uniquely positioned at the intersection of three global biodiversity hotspots (the Indo-Burma, the Himalaya, and the Mountains of Southwest China), Gaoligong harbors exceptional biodiversity shaped by its complex geological history and heterogeneous environments (Myers et al. 2000; Mittermeier et al. 2005). While botanical research in this area has traditionally focused on seed plants, significant progress has been made in documenting its bryoflora. Notably, a comprehensive survey by Ma et al. (2020) recorded 840 bryophyte species based on the examination of approximately 8,000 specimens, underscoring the region’s immense floristic richness. Despite such foundational catalogs, detailed taxonomic studies and specialized investigations into specific groups remain essential. Further in-depth research on bryophyte diversity in the Gaoligong Mountains is therefore crucial to refining our understanding of the region’s overall floristic composition and evolutionary history.

**Figure 1.**
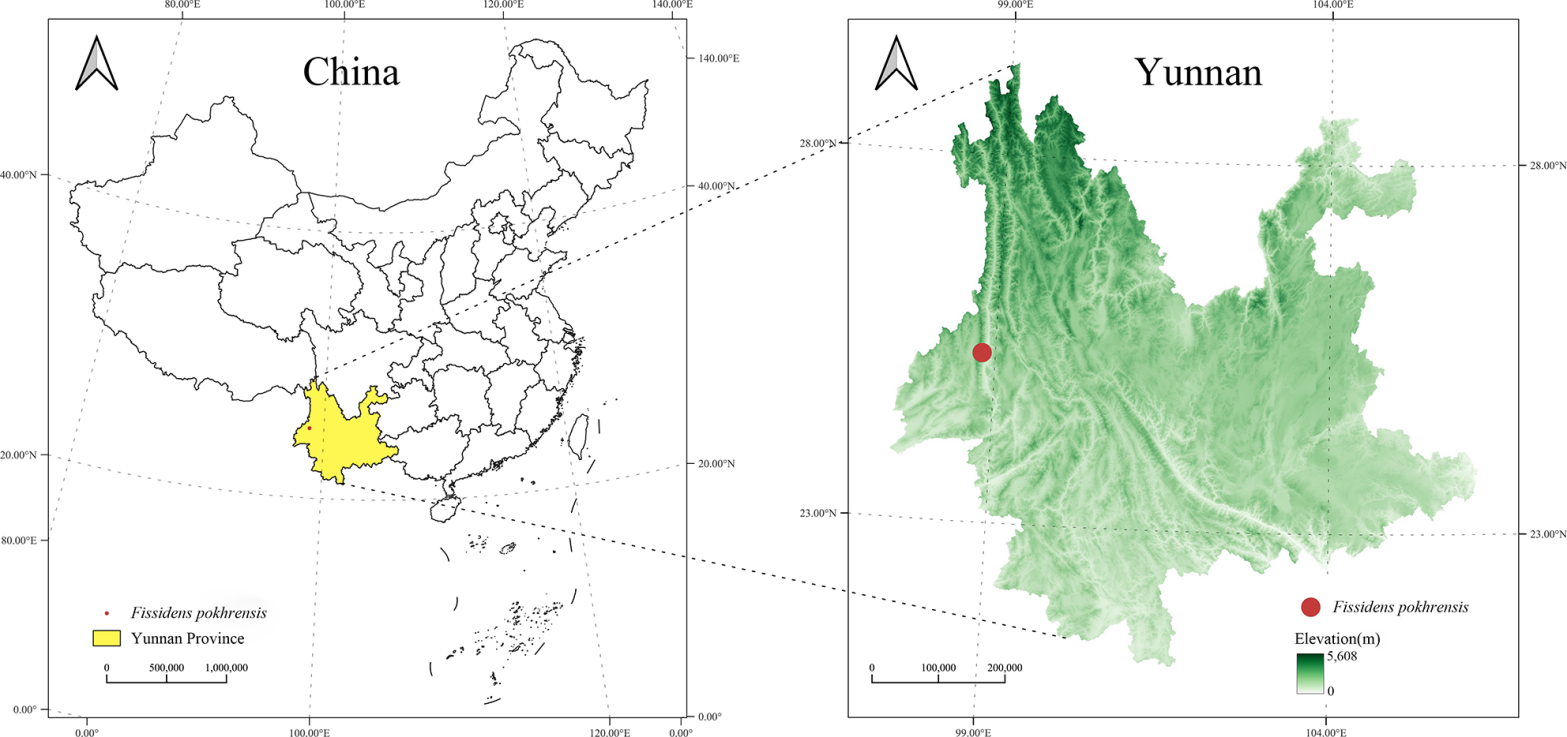
Distribution of *Fissidens pokhrensis* in Yunnan, China. Base map: National Platform for Common GeoSpatial Information Services 2025 (https://cloudcenter.tianditu.gov.cn/administrativeDivision), Approval No. GS(2024)0650 (Map compiled by An-Ran Ke).

In the process of reviewing the Chinese species of *Fissidens*, a voucher was sampled from a small population of the genus growing at the base of a tree near the Zaotang River, Baihualing, in the Gaoligong Mountains during field surveys in 2023 (Fig. 1). Morphological examination of the sample revealed a set of distinctive features that distinguished it from all documented Chinese congeners in the genus. The voucher was identified as *Fissidens pokhrensis* Nork. ex S.S.Kumar. This record represents the first documentation of the species in China.

## Materials and methods

Field images of this species were captured using a Nikon Z5 digital camera. Morphological and anatomical features were observed and documented with an Olympus SZX16 stereomicroscope and an Olympus BX53 light microscope, both equipped with an Olympus DP23 digital camera. Terminology for costa and peristome types follows Pursell and Bruggeman-Nannenga (2004) and Bruggeman-Nannenga (2021). The description of the species is based exclusively on the newly collected material, with the voucher deposited in the herbarium of Xiamen University (AU), Fujian, China.

## Results

### Fissidens pokhrensis

Taxon classification

Plantae

HypnalesLeucodontaceae

Nork. ex S.S.Kumar, Miscellanea Bryologica et Lichenologica 8(6): 120. 1979.

5D98C1E7-493D-59E2-B72E-807BA0F0DBA1

Fig. 2

#### Diagnosis.

*Fissidens pokhrensis* is characterized by its small plant size, oblong to oblong-obovate leaves with acute apices, bryoides-type costae ending 7–19 cells below the apex, pluripapillose laminal cells, and limbidia restricted to the vaginant laminae of perichaetial leaves. This species morphologically resembles *F. gardneri* Mitt. and *F. pallidinervis* Mitt. in possessing pluripapillose laminal cells and similar limbidium distribution. However, *F. pokhrensis* is distinguished by its distinctly acute leaf apices, whereas *F. gardneri* and *F. pallidinervis* typically exhibit broadly acute, obtuse, or rounded apices.

**Figure 2.**
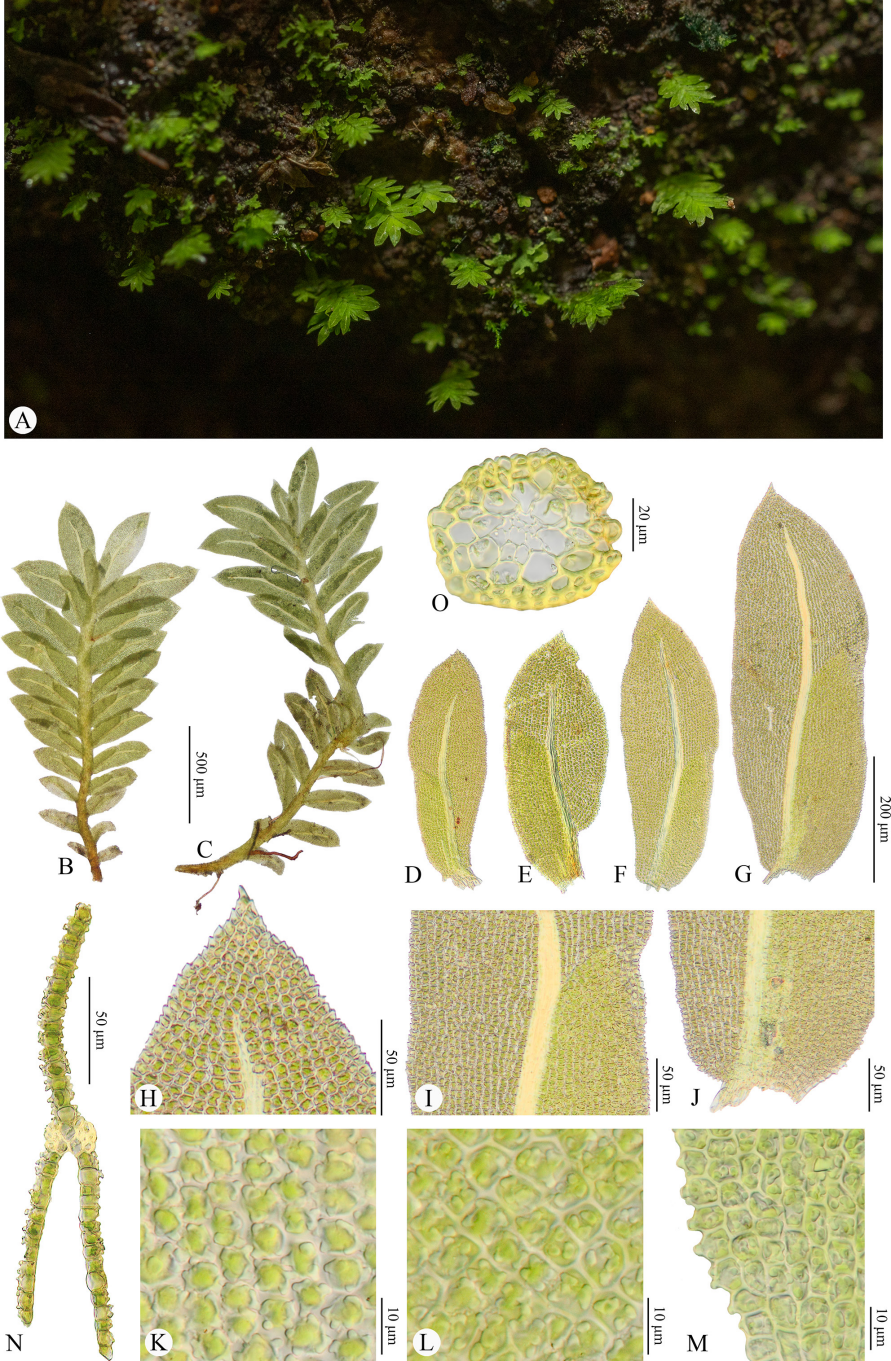
*Fissidens pokhrensis*. **A**. Habitat, wet; **B, C**. Plants; **D–G**. Leaves; **H**. Leaf apex; **I**. Median part of leaf; **J**. Leaf base; **K**. Median cells of apical lamina; **L**. Median cells of vaginant lamina; **M**. Marginal cells of vaginant lamina; **N**. Cross section of leaf; **O**. Cross section of stem. All from *W. Ye & Z.-G. Yang 20230825-03* (**A**. Taken by Wen Ye; **B–M, O**. Taken by Zi-Qi Liu; **N**. Taken by An-Ran Ke).

#### Description.

Plants small, green to yellowish green. Shoots single or occasionally branched, 2.0–2.7 mm long, 1.0–1.3 mm wide, with 8–12 pairs of leaves. Stem cortical cells in cross section small and thick-walled, central strand slightly differentiated. Axillary hyaline nodules not differentiated. Leaves curled when dry; lower ones small and remote, upper ones larger and more densely arranged, oblong to oblong-obovate, 0.6–0.8 mm long, 0.2–0.3 mm wide, apex acute; base of dorsal lamina rounded to wedge-shaped, not decurrent; vaginant laminae ca. 1/2 of leaf length, unequal; vegetative leaves elimbate; costae ending 7–19 cells below apex, bryoides-type in cross section, often slightly bent at junction of vaginant laminae; margin irregularly serrulate by projecting cells; laminal cells irregularly quadrate to hexagonal, (5–)6–8(–11) μm across, thin-walled, pluripapillose, with 4–6 papillae at the corners. Perichaetia and perigonia not seen. Sporophytes not seen.

#### Specimens examined

. China • Yunnan Province, Baoshan City, Longyang District, Mangkuan Township, Hanlong Village; 25°18.557'N, 98°48.025'E; elev. 1575 m; 25 Aug. 2023; W. Ye & Z.-G. Yang 20230825-03; on tree trunk near the tree base; AU.

#### Distribution.

New to China (Yunnan). Also known from India (Kumar 1979; Daniels and Daniel 2013; Manjula et al. 2022; Neethu et al. 2024), Japan (Suzuki 2017), and Myanmar (Bruggeman-Nannenga and Müller 2020).

#### Notes.

The Chinese specimen is identified as *F. pokhrensis* based on several key gametophytic characters consistent with the protologue (Kumar 1979). Most notably, it is small in stature, with pluripapillose laminal cells and vaginant laminae extending approximately half of the total leaf length. The leaves are oblong to oblong-obovate (described as ovate-lingulate in Kumar 1979), with the costa ending more than five cells below the acute apex. Although no perichaetial leaves are available to check for a differentiated limbidium along the vaginant laminae, the complete absence of a limbidium on all available vegetative leaves, combined with the aforementioned character states and the epiphytic habitat, provides a robust basis for this identification.

## Discussion

*Fissidens* is among the most diverse genera of mosses in China, with 65 species and 11 varieties currently documented (Redfearn et al. 1989; Li and Iwatsuki 2001; Wang et al. 2018; Ren et al. 2020; Tang et al. 2020; Chen et al. 2022; Ke et al. 2026). Most species typically inhabit rock or soil surfaces, whereas only a few are epiphytic on tree trunks. In China, approximately 11 species and two varieties of *Fissidens* are known to grow on tree trunks, including *F. anomalus* Mont., *F. crispulus* var. *robinsonii* (Broth.) Z.Iwats. & Z.H.Li, *F. dubius* P.Beauv., *F. flabellulus* Thwaites & Mitt., *F. ganguleei* Nork., *F. gardneri* Mitt., *F. gymnogynus* Besch., *F. holleanus* Dozy & Molk., *F. linearis* var. *obscuriretis* (Broth. & Paris) I.G.Stone, *Fissidens pseudoadelphinus* Z.Iwats. & Tad.Suzuki, *F. serratus* Müll.Hal., *F. teysmannianus* Dozy & Molk., and *F. virens* Thwaites & Mitt. (Li and Iwatsuki 2001; Ke et al. 2026). The newly recorded *F. pokhrensis*, which is also epiphytic, can be distinguished from these species by its oblong to oblong-obovate leaves, costa ending 7–19 cells below the leaf apex, pluripapillose laminal cells, limbidium restricted to the vaginant lamina of the perichaetial leaves, and smooth setae.

*Fissidens pokhrensis* was first validly published by Kumar (1979) based on three specimens collected from northwestern India, along the southern slopes of the Himalayas, all of which were epiphytic. Kumar noted that the margins of the species are partly limbate only in the middle part of the perichaetial leaves. Subsequently, this species was reported from Japan (Suzuki 2017), where fertile plants had narrowly lanceolate leaves—contrasting with the ovate-lingulate leaves reported in its original description. Suzuki further described it as limbate only on the vaginant laminae of upper and perichaetial leaves, with the limbidium consisting of hyaline, elongate cells in 2–3 rows. The Japanese specimens, moreover, were saxicolous, growing on a cliff. More recently, *F. pokhrensis* has also been reported from Myanmar (Bruggeman-Nannenga and Müller 2020) and the Western Ghats of India (Neethu et al. 2024), and all specimens were again collected from tree trunks. The known occurrences of *F. pokhrensis* thus span four biodiversity hotspots—the Himalaya, the Western Ghats and Sri Lanka, Indo-Burma, and Japan. Its discovery in the Gaoligong Mountains, located within the Mountains of Southwest China hotspot and adjacent to both the Indo-Burma and Himalaya hotspots (Myers et al. 2000; Mittermeier et al. 2005), is therefore biogeographically unsurprising. Given the notable variations in leaf morphology, substrate preference, and distribution patterns observed across these regions, *F. pokhrensis* exhibits a certain level of morphological and ecological variability. Consequently, further molecular and detailed morphological studies are warranted to clarify the full taxonomic limits and geographical range of this species.

*Fissidens pokhrensis* was placed in subg. *Fissidens* sect. *Semilimbidium* (Müll.Hal.) Broth. by Suzuki et al. (2018) because its limbidia are confined to the vaginant laminae of perichaetial leaves. However, Bruggeman-Nannenga (2021) demonstrated that this section, together with sections *Polypodiopsis* (Müll.Hal.) Paris, *Areofissidens* Müll.Hal., and *Aloma* (Kindb.) Tad.Suzuki & Z.Iwats., forms a clade corresponding to subg. *Aloma* (Kindb.) Pursell & Brugg.-Nann. She subsequently reinstated subg. *Polypodiopsis* (Müll.Hal.) Broth., placed subg. *Aloma* in synonymy under it, and redefined the subgenus by its bryoides-type costae, capsules with ± 32 columns of exothecial cells, and scariosus-type peristomes. At the sectional level, sect. *Antennidens* (Müll.Hal.) Paris was recognized as the senior synonym of sect. *Semilimbidium* and expanded to include sections *Semilimbidium* C.Müll., *Pycnothallia* C.Müll., *Crenularia* C.Müll., *Aloma* C.Müll. p.p. sensu Brotherus (1924), and the “*Semilimbidium* clade” of Suzuki et al. (2018) (Bruggeman-Nannenga 2022a, 2022b). This section is characterized by typically semilimbate (less often elimbate or completely limbate) leaves, small to medium-sized laminal cells that are pluripapillose, mammillose, or rarely smooth, and bryoides-type costae. Accordingly, *F. pokhrensis* is here assigned to subg. *Polypodiopsis* sect. *Antennidens*.

The following updated key is provided for the Chinese species of *Fissidens* characterized by small to medium-sized plants, mammillose or papillose laminal cells, and a limbidium restricted to the vaginant laminae, ranging from being present on all leaves to being restricted solely to the perichaetial leaves.

### Key

**Table:** 

1	Laminal cells mammillose or unipapillose	**2**
–	Laminal cells pluripapillose	**8**
2	Vaginant laminae ca. 3/4 of total leaf length; exothecial cells mammillose to distinctly unipapillose	***F. capitulatus***
–	Vaginant laminae usually less than 3/4 of total leaf length; exothecial cells smooth	**3**
3	Leaf apices more or less obtuse; costae ceasing below apices	***F. rupicola***
–	Leaf apices acute to narrowly acute; costae percurrent to excurrent	**4**
4	Margin of vaginant laminae distinctly serrate	***F. serratus***
–	Margin of vaginant laminae almost entire to serrulate or crenulate	**5**
5	Laminal cells with one tiny papilla	***F. schwabei***
–	Laminal cells distinctly mammillose or papillose	**6**
6	Laminal cells unipapillose; costae excurrent; central strand weakly differentiated	***F. virens***
–	Laminal cells highly mammillose and usually with one (rarely two) papilla; costae percurrent to shortly excurrent; central strand not differentiated	**7**
7	Plants cladautoicous	***F. crenulatus* var. crenulatus**
–	Plants rhizoautoicous	***F. crenulatus* var. *pursellii***
8	Leaves densely overlapping; upper leaves narrowly lanceolate or linear-lanceolate	**9**
–	Leaves laxly arranged; upper leaves oblong-lanceolate to lanceolate, oblong-lingulate, or oblong to oblong-obovate	**11**
9	Limbidia weak, found only on lower half of vaginant laminae of upper and perichaetial leaves	***F. linearis* var. *obscuriretis***
–	Limbidia distinct, found on vaginant laminae of almost all leaves	**10**
10	Limbidia restricted to lower half of vaginant lamina	***F. wichurae***
–	Limbidia differentiated along the entire margin of vaginant laminae	***F. kinabaluensis***
11	Laminal cells highly mammillose, with 2–5 high papillae clustered at the center of each cell lumen	***F. incognitus***
–	Laminal cells pluripapillose, papillae not clustered at the center of each cell lumen	**12**
12	Limbidia present on vaginant laminae of almost all leaves	**13**
–	Limbidia restricted to vaginant laminae of perichaetial leaves	**14**
13	Limbidia always marginal	***F. holleanus***
–	Limbidia often intramarginal by one or two rows of laminal cells proximally	***F. ceylonensis***
14	Leaf apices acute	***F. pokhrensis***
–	Leaf apices broadly acute to obtuse to rounded	**15**
15	Plants 0.5–3.5 mm tall; leaves short-oblong-lingulate, 0.3–0.8 mm long; peristome anomalous (reduced)	***F. gardneri***
–	Plants to 9 mm tall; leaves oblong to lanceolate, to 1.2 mm long; peristome of *scariosus*-type	***F. pallidinervis***

## Supplementary Material

XML Treatment for Fissidens pokhrensis

## Additional information

### Artificial Intelligence (AI) use

The authors accept full responsibility for the content of the manuscript, including the disclosure of any use of AI.

No AI tools were used in the preparation of this manuscript.

### Author ORCIDs

Z.-Q. Liu https://orcid.org/0009-0004-3455-2468

A.-R. Ke https://orcid.org/0009-0007-5981-0381

B.-C. Ho https://orcid.org/0000-0003-0530-775X

S.-L. Wang https://orcid.org/0009-0003-4719-0286

Y.-M. Wei https://orcid.org/0000-0002-1845-716X

W. Ye https://orcid.org/0000-0002-5956-800X

### Data availability

All of the data that support the findings of this study are available in the main text.
